# Fungal Diversity Drives Non-Linear Trajectories of Soil Multifunctionality During Alpine Grassland Restoration

**DOI:** 10.3390/microorganisms14030562

**Published:** 2026-03-01

**Authors:** Minghui Meng, Jiakai Shi, Sha Zhou, Danni Peng, Yihan Fu, Mengmeng Wen, Jun Wang, Fazhu Zhao

**Affiliations:** 1 College of Urban and Environmental Sciences, Northwest University, Xi’an 710127, China15340952795@163.com (M.W.);; 2 Shaanxi Key Laboratory of Earth Surface System and Environmental Carrying Capacity, Northwest University, Xi’an 710127, China; 3 Xi’an Botanical Garden of Shaanxi Province (Institute of Botany of Shaanxi Province), Xi’an 710061, China; 4 Carbon Neutrality College (Yulin), Northwest University Xi’an, Xi’an 719053, China; 5 State Key Laboratory of Soil Erosion and Dryland Farming on the Loess Plateau, Institute of Soil and Water Conservation, Chinese Academy of Sciences and Ministry of Water Resources, Yangling 712100, China; 6 Shaanxi Xi’an Urban Ecosystem National Observation and Research Station, National Forestry and Grassland Administration, Xi’an 710127, China

**Keywords:** alpine grassland, soil multifunctionality, fungal diversity, restoration chronosequence

## Abstract

Despite the widely recognized importance of grassland restoration for soil multifunctionality (SMF), its temporal dynamics along the restoration chronosequence and the relative contributions of bacterial and fungal diversity to SMF remain poorly understood, particularly in alpine grasslands. Here, we examined SMF along an alpine grassland restoration chronosequence (1, 5, 7, 13, and 20 years) on the Qinghai–Tibet Plateau. We found that SMF exhibited a pronounced non-linear trajectory, increasing by 39.13% from year 1 to year 7, subsequently declining by 50% and 46.88% at years 13 and 20, respectively, relative to the peak at year 7. Fungal richness varied markedly across the restoration chronosequence, peaking in year 5 with a 16.03% increase relative to year 1, and was positively associated with SMF, whereas bacterial richness showed no significant relationship. Structural equation modeling further confirmed that, along with soil moisture, fungal richness was significantly associated with SMF. Together, our findings highlight fungal diversity as a key driver of SMF during alpine grassland restoration and improve process-based predictions of alpine grassland functioning under ongoing climate change.

## 1. Introduction

Grasslands are one of the most widespread terrestrial ecosystems, playing indispensable roles in global biogeochemical cycles, energy flows, and ecosystem functions [1,2]. As the world’s highest-altitude grassland ecosystem, alpine meadows on the Qinghai–Tibet Plateau (QTP) play a critical role in regional ecological security and global change regulation [3]. However, most meadow soil on the QTP has been degraded by continuous grazing, substantially impairing SMF [3,4]. It is widely recognized that ecological restoration, whether natural or artificial, represents an effective strategy for mitigating biodiversity loss and enhancing ecosystem multifunctionality [5,6]. Nevertheless, numerous studies have examined grassland changes across different degrees of degradation [3,7], but investigations into the dynamics of SMF along the restoration chronosequence and the underlying microbial drivers remain poorly understood. Addressing this gap is essential for enhancing predictions of ecosystem responses to anthropogenic disturbances and future global change.

Grassland restoration has been demonstrated to enhance SMF through the recovery of belowground biodiversity in concert with improvements in soil abiotic conditions [8]. For instance, an assessment of a 22-year grassland restoration project revealed that SMF increased with restoration duration [9]. During the early stages of restoration, pronounced shifts in aboveground plant cover and biomass occur, with cascading effects on microbial community structure and nutrient cycling [10]. In contrast, gains in plant diversity diminish and approach saturation as restoration progresses [10,11]. Beyond this threshold, soil nutrient accumulation may become constrained [8], such that changes in SMF along the restoration chronosequence are unlikely to increase monotonically. Therefore, understanding how SMF responds along the restoration chronosequence is critical.

Soil ecosystems sustain multiple functions simultaneously, a fundamental principle of soil science recognized since the era of V. Dokuchaev [12,13]. In contemporary research, this inherent complexity is addressed through SMF indices, which have recently been employed to quantitatively assess the collective performance of potential soil functions [2,3]. These multifaceted processes are largely mediated by the belowground microbial community [14]. Bacteria and fungi play pivotal roles in maintaining soil functions owing to their involvement in elemental biogeochemical cycling [15]. However, the relative contributions of bacterial and fungal diversity to SMF during vegetation restoration remain controversial. For instance, studies in sandy land restoration have shown that soil bacterial diversity exerts a significantly stronger positive effect on SMF than fungal diversity [16]. By contrast, SMF in boreal forests is primarily governed by fungal diversity [17]. Although both fungal and bacterial diversity are recognized as important contributors to SMF, their functional significance may shift in response to environmental change. Notably, fungi generally exhibit greater tolerance to environmental stresses such as low temperature and drought [2,18]. In addition, as vegetation recovers, changes in aboveground vegetation cover and biomass often occur [10]. Fungi often form extensive symbiotic relationships with plants and dominate the decomposition of recalcitrant carbon compounds [3]. Therefore, fungal diversity is predicted to play a leading role in regulating SMF in alpine grasslands.

To address the above issues, we conducted a study along an alpine grassland restoration chronosequence (1, 5, 7, 13, and 20 years) on the Qinghai–Tibet Plateau. The objectives were to characterize the dynamics of SMF across the restoration chronosequence, as well as the relationships between fungal and bacterial diversity and SMF. Given that gains in soil resources and ecosystem processes during grassland restoration are not sustained indefinitely but tend to saturate at later stages, we hypothesized that (1) soil multifunctionality would exhibit a non-linear trajectory along the restoration chronosequence. Furthermore, considering that fungi exhibit greater adaptability to low-temperature and drought stresses in alpine environments and are more efficient at decomposing recalcitrant carbon during grassland restoration, we hypothesized that (2) fungal diversity, rather than bacterial diversity, would drive soil multifunctionality in alpine grasslands. By clarifying the temporal patterns and microbial drivers of SMF, this study provides mechanistic insights into belowground controls on ecosystem recovery and informs alpine grassland restoration.

## 2. Materials and Methods

### 2.1. Study Area and Field Sampling

**Figure 1:**
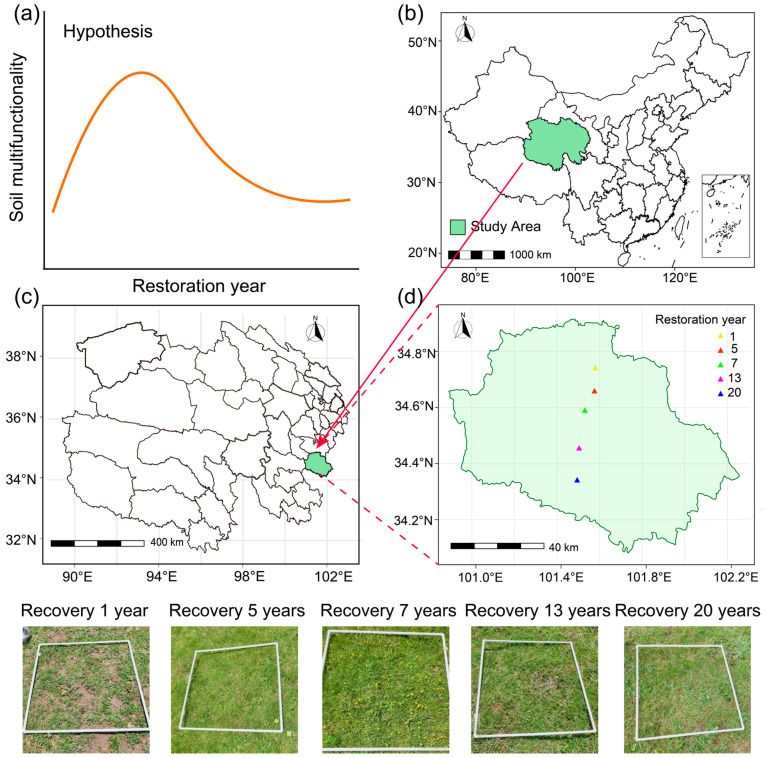
Conceptual framework and sampling site distribution: (**a**) the theoretical hypothesis of this study; (**b**) location of the study area within China; (**c**) geographical location of the study area on the Qinghai–Tibet Plateau; and (**d**) spatial distribution of sampling sites within the study area.

Field experiments were conducted in a typical alpine meadow on the eastern Qinghai–Tibet Plateau (101°20′–101°35′ E, 34°20′–34°44′ N), with an elevation ranging from 3477 to 3611 m. The sampling site locations are illustrated in Figure 1. The region experiences a plateau continental climate, characterized by a mean annual temperature of 0.2–1.7 °C and a mean annual precipitation of 639.7–696.1 mm, with no absolute frost-free season. To minimize the potential influence of slope preferential flow and water redistribution, all sampling sites were established on plots with near-identical topographical features (slope < 5°, similar aspect and elevation). The vegetation is dominated by species of *Poaceae* and *Cyperaceae*, accompanied by various forbs, with the dominant plant species including *Kobresia pygmaea*, *Oxytrophic ochrocephala*, *Carex moorcroftii*, and *Elsholtzia densa Benth*, among others [2]. A detailed plant species list is provided in Table S1. The soil in the study area is classified as alpine meadow soil (Chinese Soil Taxonomic System), which corresponds to Cambisols according to the World Reference Base for Soil Resources (WRB 2022), and exhibits a typical O-Ah-Bw-C horizon sequence, consisting of a surface organic layer (O), a humic mineral topsoil (Ah), a weakly developed cambic horizon (Bw), and an underlying parent material (C) [2,19].

Field sampling was carried out in June 2025 along a restoration chronosequence (1, 5, 7, 13, and 20 years). Sites were selected based on similar topographical features to minimize environmental heterogeneity. Six replicate plots (10 m × 10 m) were established at each site. Prior to sampling, the surface organic layer (O horizon), composed of fresh and partially decomposed litter, was carefully removed. Five soil cores were then randomly collected from the exposed topsoil (0–10 cm, Ah horizon) and combined to form a composite sample. Any remaining visible roots and litter were subsequently removed. The drilling method was prioritized to minimize ecological disturbance in this fragile alpine ecosystem while ensuring statistical representativeness through multi-point composite sampling [3]. This depth was selected because the topsoil layer represents the most biologically active zone, where rapid changes in microbial communities and nutrient dynamics occur during grassland restoration [10]. This yielded a total of 30 soil samples. Each composite sample was divided into three subsamples: one was air-dried for physicochemical analysis, the second was stored at 4 °C for microbial biomass and enzyme activity assays, and the third was stored at −80 °C for DNA extraction and amplicon sequencing.

### 2.2. Soil Physicochemical Analyses

Soil pH was determined using a pH meter at a soil-to-water ratio of 1:2.5, while soil water content (SWC) was measured gravimetrically. Particle-size fractionation was conducted by dispersing soil samples with sodium hexametaphosphate and passing the suspension through a 53 μm sieve. Material retained on the sieve was classified as particulate organic matter (POM), whereas the filtrate was further separated into mineral-associated organic matter (MAOM). Particulate organic carbon (POC) and mineral-associated organic carbon (MAOC), representing the organic carbon in the respective fractions, were quantified with an elemental analyzer. Soil organic carbon (SOC) was quantified via potassium dichromate oxidation [20], total nitrogen (TN) was measured using the Kjeldahl method, and total phosphorus (TP) was quantified by the molybdenum–antimony colorimetric method. Microbial biomass carbon (MBC), nitrogen (MBN), and phosphorus (MBP) were assessed using the chloroform fumigation–extraction technique with extraction coefficients of 0.45, 0.50, and 0.40, respectively [21]. Extracellular enzyme activities were quantified following standard fluorometric assays [22], including C-acquiring enzymes (β-glucosidase, cellobiohydrolase), N-acquiring enzymes (β-1,4-N-acetylglucosaminidase, leucine aminopeptidase), and P-acquiring enzymes (acid phosphatase). Mean annual temperature (MAT) and mean annual precipitation (MAP) data were obtained from the National Earth System Science Data Center, National Science and Technology Infrastructure of China (http://www.geodata.cn, accessed on 25 November 2025). Within each 10 m × 10 m plot, three 1 m × 1 m PVC quadrats were deployed, and vegetation coverage was determined via visual estimation as the percentage of the quadrat area covered by vascular plant vertical projection, with the mean of three replicates used for analysis.

### 2.3. DNA Extraction, Sequencing, and Analysis of Soil Microbial Richness

Total genomic DNA was extracted using commercial kits, with concentration and purity assessed on a NanoDrop One spectrophotometer (Thermo Fisher Scientific, Waltham, MA, USA) [23]. The hypervariable regions of microbial rRNA genes were amplified via PCR using barcoded primers targeting the bacterial 16S V4 region (515F/806R) and the fungal ITS region. PCR reactions were conducted under the following conditions: 94 °C for 5 min; 30 cycles of 94 °C for 30 s, 52 °C for 30 s, and 72 °C for 30 s; and a final extension at 72 °C for 10 min. Amplicons were verified using the E.Z.N.A.^®^ Gel Extraction Kit (Omega Bio-tek, Norcross, GA, USA) and quantified prior to library construction. Sequencing libraries were prepared with the NEBNext^®^ Ultra™ II DNA Library Prep Kit (Findrop/Magigene, Guangzhou, China) for Illumina and sequenced on an Illumina Nova 6000 platform (Guangdong Magigene Biotechnology Co., Ltd., Guangzhou, China). Raw paired-end reads were quality-filtered and adapter-trimmed using fastq (version 0.14.1) and cutadapt (https://github.com/marcelm/cutadapt/, accessed on 25 February 2026). and then merged with USEARCH (version 10). Amplicon sequence variants (ASVs) were obtained through error correction and denoising with the UNOISE3 algorithm in USEARCH, followed by chimera removal. Taxonomy was assigned to representative ASVs using the USEARCH-SINTAX algorithm against the SILVA and UNITE databases at a confidence threshold of 0.8 [24]. ASVs classified as chloroplast, mitochondrial, or unclassified at the kingdom level were removed, yielding a final ASV abundance table for downstream analysis. To minimize biases caused by unequal sequencing depth, ASV abundance tables were rarefied to a consistent sequencing depth across samples [17].

### 2.4. Quantification of Soil Multifunctionality

We screened an initial set of 19 functional indicators using Spearman’s rank correlation analysis. Indicators with pairwise correlation coefficients ≥0.7 were excluded to avoid multicollinearity, yielding a final set of 14 variables (Table S2). These encompassed metrics of C cycling (POC, MAOC, and DOC), N cycling (NH4+-N, DON), P cycling (TP, AP), microbially mediated nutrient turnover (extracellular enzymes: PER, NAG, PPO, and URE), and microbial biomass (MBC, MBN, and MBP), which were collectively used to assess soil multifunctionality [6,25]. All functional indicators were normalized to a consistent range (0–1) using the min–max standardization method, and ecosystem multifunctionality was calculated as the average of the standardized indicators [26]. This index represents an operational proxy of soil functional performance widely used in ecosystem-function studies, rather than a direct measurement of soil ecological functions as defined in classical soil science theory. We employed a multi-threshold approach to assess the relationship between species richness and SMF across a continuum of function thresholds [27]. Thresholds were systematically evaluated from 5% to 99% at 1% increments, allowing us to account for potential functional trade-offs and comprehensively quantify biodiversity–SMF relationships.

### 2.5. Statistical Analyses

All statistical analyses were performed using R (4.5.1). Microbial α-diversity was evaluated by calculating microbial richness using the “vegan” R package [28]. To evaluate the impact of restoration duration on soil multifunctionality and microbial diversity, one-way ANOVA followed by LSD multiple tests was conducted using the “agricolae” package in R. Ordinary least squares (OLS) regressions were conducted to explore the relationships between SMF and soil microbial diversity. Structural equation modeling (SEM) was performed to disentangle the direct and indirect effects of climate (MAT and MAP), soil properties (pH, soil moisture), and microbial diversity (bacterial and fungal richness) on soil multifunctionality. This approach allowed us to assess whether the associations between microbial diversity and SMF persisted after accounting for multiple covarying environmental factors. The conceptual models were evaluated using the “piecewise SEM” package. Variables showing significant bivariate relationships with multifunctionality were incorporated into the initial structural equation models. In addition, random forest modeling (RFM) was used to identify the most important fungal taxa affecting SMF.

## 3. Results

### 3.1. Soil Multifunctionality Along the Grassland Restoration Chronosequence

**Figure 2:**
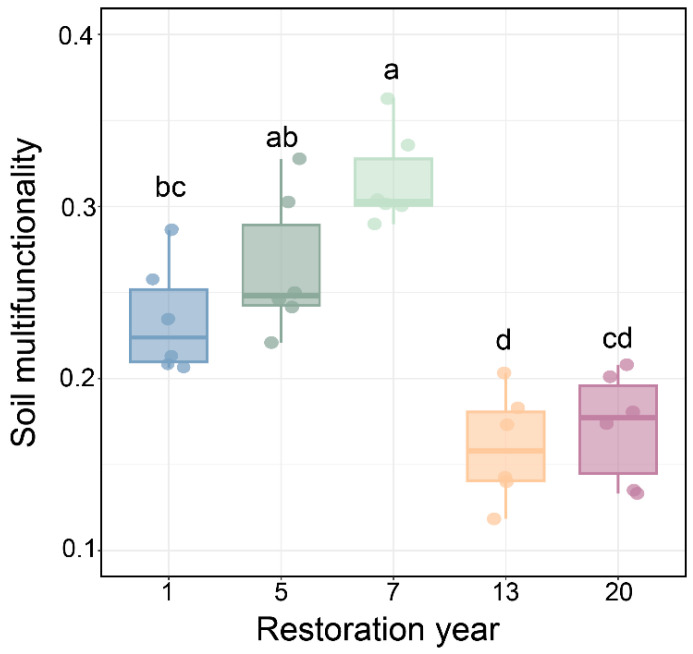
Soil multifunctionality across the restoration chronosequence in alpine grasslands. Different letters indicate significant differences among restoration durations (one-way ANOVA followed by LSD, *p* < 0.05). Data are means ± SD (*n* = 6).

Along the grassland restoration chronosequence, SMF displayed a unimodal pattern, characterized by an initial increase followed by a decrease and eventual stabilization (Figure 2). Specifically, SMF increased from 0.23 at year 1 to 0.26 at year 5, reached a maximum of 0.32 at year 7, and subsequently declined significantly to 0.16 and 0.17 at years 13 and 20, respectively. Although SMF at year 5 did not differ significantly from that at 1 or 7 years, it was significantly higher than the values observed at 13 and 20 years. During the long-term restoration stage (13 and 20 years), SMF remained at a relatively stable yet significantly lower level compared to the peak at year 7, with no significant difference observed between the two stages.

### 3.2. Variations in Soil Microbial Diversity and Their Effects on SMF

**Figure 3:**
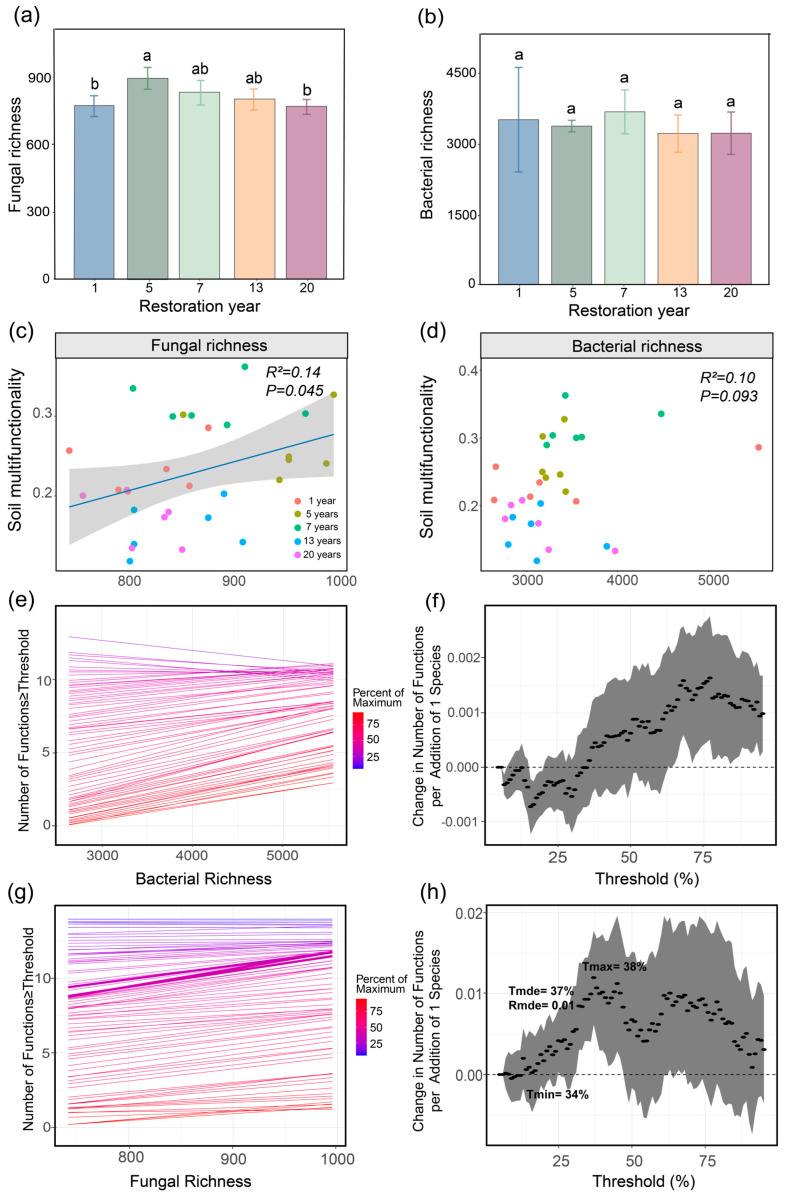
Relationships between soil multifunctionality and microbial diversity. Changes in fungal (**a**) and bacterial richness (**b**) across the restoration chronosequence (different lowercase letters indicate significant differences at *p* < 0.05). Ordinary least squares (OLS) regression between soil multifunctionality and fungal (**c**) or bacterial richness (**d**). Effects of bacterial (**e**) and fungal (**g**) richness on the number of functions above thresholds. Lines represent the slope of the relationship between soil microbial richness and the number of functions exceeding threshold values ranging from 5% to 95% of their respective maximum values. The dotted curves represent the rate of change in the number of functions per unit increase in bacterial (**f**) and fungal (**h**) richness.

Our results revealed that only fungal richness differed significantly across the grassland restoration chronosequence (Figure 3a), whereas bacterial richness showed no significant change (Figure 3b). Fungal richness was highest in year 5 of restoration, representing a 16.03% increase compared to year 1 and being 16.56% higher than that observed in year 20 (*p* < 0.05). Meanwhile, changes in fungal richness had a considerable impact on SMF. The OLS linear regression analysis indicated that fungal richness was significantly positively correlated with SMF (Figure 3c). Using a multiple-threshold approach, we further found that the effect of fungal richness on SMF exhibited distinct threshold responses. Specifically, the minimum threshold (Tmin) occurred at 34%, the relationship peaked at the median threshold (Tmde = 37%) with a slope of 0.01 (Rmde), and the maximum threshold (Tmax) was observed at 38% (Figure 3g,h).

Based on the finding that fungal richness was the only significant microbial predictor of SMF, we further explored the associations between fungal genera and SMF, with a total of 400 fungal genera included in these analyses. *Mortierella* was the dominant genus in the fungal community with an average relative abundance of nearly 30% across all samples (Figure S1). Fifteen fungal genera were identified as the most important predictors of SMF, and this result was further corroborated by Spearman’s correlation analyses (Figure S2). To infer the functional attributes of these key genera, functional guilds were assigned using FUNGuild based on taxonomic information. Among these genera, *Tausonia*, *Solicoccozyma*, *Gibberella*, *Dactylonectria*, and *Exophiala* were consistently identified as important taxa linked to SMF. Notably, the majority of fungal functional groups strongly associated with SMF were identified as saprotrophs.

### 3.3. Possible Drivers of Soil Multifunctionality

**Figure 4:**
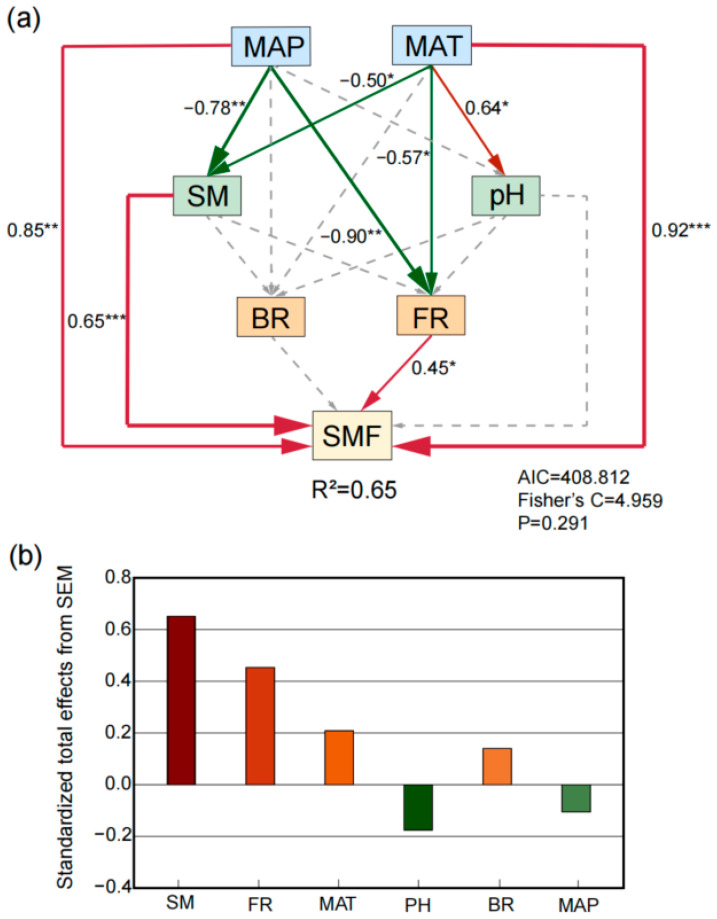
Structural equation model (SEM) analyzing the drivers of soil multifunctionality (**a**); standardized total effects derived from the SEM (**b**). MAT, mean annual temperature; MAP, mean annual precipitation; SM, soil moisture; BR, bacterial richness; FR, fungal richness; SMF, soil multifunctionality. Red and green arrows represent significant positive and negative pathways, respectively, while gray dashed arrows represent non-significant pathways. Arrow width corresponds to the standardized path coefficient. Significance levels of each predictor are indicated by asterisks; * *p* < 0.05, ** *p* < 0.01, *** *p* < 0.001. R^2^ values denote variance explained.

We employed SEM to elucidate the mechanisms underlying the effects of grassland restoration duration on SMF (Figure 4 and Figure S3). The results showed that MAT, MAP, soil moisture, and fungal richness directly and indirectly influenced SMF (Figure 4a). MAT and MAP affected SMF through both direct pathways and indirect pathways mediated by fungal richness. Fungal diversity had a significant mediating effect on SMF, whereas bacterial diversity did not. Furthermore, MAP and MAT exerted significant indirect effects on SMF through their regulation of SM, with SM showing a significant positive relationship with SMF. Additionally, for SMF across different restoration durations, SM, fungal richness, MAT, soil pH, bacterial richness, and MAP had standardized total effects of 0.65, 0.45, 0.21, −0.18, 0.14, and −0.11, respectively (Figure 4b). Considering both direct and indirect effects, soil moisture was the most important abiotic factor positively influencing SMF, followed by fungal richness.

## 4. Discussion

### 4.1. Non-Linear Restoration Patterns of Soil Multifunctionality Along the Chronosequence

Consistent with our hypothesis, SMF displayed a hump-shaped pattern along the grassland restoration chronosequence (Figure 2), indicating that SMF does not increase monotonically with restoration duration but instead reflects stage-dependent coupling between soil resource availability and biotic processes. In the initial restoration stages, grazing exclusion alleviated soil compaction caused by herbivory, thereby improving soil pore structure and infiltration capacity and increasing soil moisture (Table S3) [25,29]. Increased soil moisture was closely linked to elevated vegetation cover (from 74.67% to 98.83% at year 7, Table S3) and improved nutrient availability [30,31]. In turn, increased SOC and TN contributed substantially to regulating enzyme diffusion rates, substrate availability, and microbial community structure, ultimately promoting SMF (Figure S5) [32]. By year 7 of restoration, peak soil moisture and nutrient levels coincided with increased vegetation cover, fostering substrate supply and optimized soil C:N stoichiometry that supported the coordinated expression of multiple soil functions [33]. In contrast, during later restoration stages, ecosystem processes tended to stabilize, vegetation coverage remained at a high and stable level, while soil moisture declined, potentially due to higher litter interception, and net SOC and TN accumulation weakened [31]. Under such resource-limited conditions, further improvements in SMF were constrained.

### 4.2. Variations in Bacterial and Fungal Diversity Along the Restoration Chronosequence

Fungal diversity varied significantly along the grassland restoration chronosequence, whereas bacterial diversity remained relatively stable (Figure 3a,b), indicating that fungal communities underwent succession during grassland restoration. Although moderate increases in MAP and MAT have been reported to enhance microbial activity [34], and they are also known to promote soil fungal diversity [35,36], such responses can be divergent or even reversed under extreme alpine conditions (Figure 4). On the Qinghai–Tibet Plateau, rising temperatures can induce soil drying, thereby limiting the dispersal and metabolic activity of saprotrophic fungi [37]. In addition, due to the loose soil structure and low water-holding capacity of alpine grasslands, increased precipitation does not necessarily lead to higher soil moisture, and excessive precipitation results in a reduction in soil oxygen availability, creating microsites that are unfavorable for fungal persistence [38]. Consistently, large-scale studies have shown that fungal richness, particularly of ectomycorrhizal fungi, often peaks in cold and dry environments [35], reflecting the dominance of cold-adapted and stress-tolerant taxa. Overall, climate plays a key role in regulating fungal diversity during grassland restoration on the Qinghai–Tibet Plateau.

### 4.3. Fungal Diversity Drives Soil Multifunctionality

Gaining a full understanding of these complex dynamics is difficult without recognizing the pivotal role that microorganisms play in regulating terrestrial biogeochemical cycles [32]. Interestingly, we found a positive relationship between fungal diversity and SMF, which was further supported by SEM results showing a positive effect of fungal diversity on SMF. This finding is consistent with previous studies. For example, Ma et al. [39] demonstrated that SMF was positively driven by soil fungal diversity but not by bacterial diversity. This observation may be explained by several mechanisms. First, microbial necromass constitutes a major component of soil organic matter [40], with fungal necromass contributing more substantially than bacterial necromass in grassland ecosystems (Figure S6). Second, this could be attributed to the different carbon utilization strategies between those two decomposers [3]. Fungi are efficient in decomposing recalcitrant carbon compounds (e.g., cellulose and lignin) [3,17] and generally prefer substrates with higher C:N ratios in comparison to bacteria [41]. In our study, the SOC:TN ratio increased significantly during the mid-restoration stage (Table S3). Thus, our study highlights that fungal diversity is essential for regulating SMF, implying that its loss may reduce the functional resilience of restored grasslands.

As fungi made a disproportionate contribution to SMF, we identified several saprotrophic fungal taxa, including *Tausonia* and *Solicoccozyma*, as potentially key regulators of soil functions in alpine grasslands (Figure S2). These fungi are rapid responders to newly available organic matter [42] and efficiently decompose both labile and complex carbon substrates through extensive hyphal networks and high extracellular enzyme activities [43], thereby accelerating soil carbon turnover and nutrient release [44,45]. However, most predictor fungi are typical r-strategy yeasts that tend to exploit labile resources [17], and their dominance could disproportionately enhance decomposition-related processes at the expense of other soil functions [46]. Such a scenario could reduce functional complementarity and ultimately constrain the overall enhancement of soil multifunctionality.

Microbial co-occurrence networks elucidate interactions among taxa and their contributions to soil multifunctionality [14,47]. During grassland restoration, both the number of edges and nodes in fungal co-occurrence networks changed significantly (Table S4), but SMF was not significantly correlated with soil fungal network complexity (Figure S7). This pattern is likely attributable to functional redundancy, whereby multiple species perform similar functional roles, thereby reducing the dependence of ecosystem functioning on complex network associations [42]. Functional redundancy is widespread in soil microbes [6]. These results further indicate that changes in SMF during grassland restoration on the Qinghai–Tibet Plateau are primarily driven by fungal diversity.

### 4.4. Limitation

A primary limitation of this study is its lack of a comprehensive pedological framework that integrates soil genesis and morphology. By focusing on the 0–10 cm humic horizon, this research may have overlooked functional shifts to deeper profiles as root systems develop. Consequently, the observed decline in surface soil multifunctionality might represent a vertical redistribution of functions within the soil architecture rather than a systemic loss. Furthermore, while vegetation coverage was recorded, the species richness of *Poaceae* and *Cyperaceae* was not explicitly determined, hindering a direct quantification of the linkage between specific plant diversity and fungal diversity. Future research should integrate profile-scale assessments with metagenomic or metatranscriptomic approaches to better correlate high-throughput microbial data with actual pedogenic processes occurring across the soil profile.

## 5. Conclusions

Our study shows that soil multifunctionality exhibits a non-linear trajectory along the grassland restoration chronosequence on the Qinghai–Tibet Plateau, characterized by an initial increase followed by a subsequent decline. We further show that fungal diversity varies markedly across the chronosequence and is positively associated with SMF, whereas bacterial diversity shows no significant influence. In addition, soil moisture emerged as a key abiotic regulator of SMF, underscoring the role of hydrological conditions in shaping SMF. Overall, our findings highlight that fungal diversity, in concert with soil moisture, plays a crucial role in maintaining SMF during alpine grassland restoration. These insights advance the mechanistic understanding of belowground drivers and improve predictions of alpine grassland functioning under ongoing climate change.

## Data Availability

All the sequencing data have been deposited on the National Center for Biotechnology Information website (SRP669864).

## Supplementary Materials

The following supporting information can be downloaded at: https://www.mdpi.com/article/10.3390/microorganisms14030562/s1, Figure S1: Relative abundance of fungal taxa across the restoration chronosequence. Figure S2: Importance of fungal taxa at the genus level identified as predictors of soil multifunctionality using random forest modeling. Panel (a) shows the mean predictor importance (% of increase in MSE) of fungal genera on soil multifunctionality, based on the random forest model. Panel (b) shows the correlations between the significant fungal genera and soil multifunctionality. Significance levels are indicated as follows: * *p* < 0.05; ** *p* < 0.01; *** *p* < 0.001. Figure S3: A priori structural equation modeling (SEM) metamodel evaluating the drivers of soil multifunctionality. Figure S4: Correlations between soil moisture and soil organic carbon (a) and total nitrogen (b). Figure S5: Correlations between soil multifunctionality and soil organic carbon (a) and total nitrogen (b). Figure S6: Differences in fungal and bacterial necromass carbon. Significant differences are indicated by asterisks *** *p* < 0.001. Figure S7: Correlations between soil multifunctionality and fungal (a) or bacterial complexity (b). Table S1: Dominant plant species in the alpine grassland study area. Table S2: Spearman correlations among soil variables used to calculate the soil multifunctionality index. Correlation coefficients and their significance levels are shown. * *p* < 0.05, ** *p* < 0.01 and *** *p* < 0.001. Table S3: The basic soil properties during grassland restoration. Different letters indicate significant differences among stages (one-way ANOVA followed by LSD, *p* < 0.05). Values are means ± SD (n=6). Table S4: Topological properties of fungal co-occurrence networks across the restoration chronosequence. Different letters indicate significant differences among stages (one-way ANOVA followed by LSD, *p* < 0.05). Values are means ± SD (n=6).

## References

[1] Wang Y., Sun J., Lee T.M. (2023). Altitude dependence of alpine grassland ecosystem multifunctionality across the Tibetan Plateau. J. Environ. Manag..

[2] Duan C., Li X., Li C., Yang P., Chai Y., Xu W. (2023). Positive effects of fungal β diversity on soil multifunctionality mediated by pH in the natural restoration succession stages of alpine meadow patches. Ecol. Indic..

[3] Wang J., Wang X., Liu G., Zhang C., Wang G. (2021). Bacterial richness is negatively related to potential soil multifunctionality in a degraded alpine meadow. Ecol. Indic..

[4] Yang F., Niu K., Collins C.G., Yan X., Ji Y., Ling N., Zhou X., Du G., Guo H., Hu S. (2018). Grazing practices affect the soil microbial community composition in a Tibetan alpine meadow. Land Degrad. Dev..

[5] Kang Y., Shen L., Li C., Huang Y., Chen L. (2025). Increased soil multifunctionality is determined by altered bacterial–fungal–protistan compositions and co-occurrence network complexity during vegetation restoration in a Karst region, southwest China. J. Appl. Ecol..

[6] Chen Y., Chi J., Lu X., Cai Y., Jiang H., Zhang Q., Zhang K. (2023). Fungal-bacterial composition and network complexity determine soil multifunctionality during ecological restoration. Catena.

[7] Zhou H., Zhang D., Jiang Z., Sun P., Xiao H., Wu Y., Chen J. (2019). Changes in the soil microbial communities of alpine steppe at Qinghai-Tibetan Plateau under different degradation levels. Sci. Total Environ..

[8] Xu H., Chen C., Pang Z., Zhang G., Zhang W., Kan H. (2025). Compared with plant diversity and soil fungal diversity, soil bacterial diversity drives ecosystem multifunctionality during the vegetation restoration process. Appl. Soil Ecol..

[9] Resch M.C., Schütz M., Buchmann N., Frey B., Graf U., van der Putten W.H., Zimmermann S., Risch A.C. (2021). Evaluating long-term success in grassland restoration: An ecosystem multifunctionality approach. Ecol. Appl..

[10] Liu M., He W., Zhang Z., Sun J., Cong N., Nie X., Wang Y., Zhang L., Yang B., Chen Y. (2022). Mutual feedback between above-and below-ground controls the restoration of alpine ecosystem multifunctionality in long-term grazing exclusion. J. Clean. Prod..

[11] Guo Y., Hou L., Zhang Z., Zhang J., Cheng J., Wei G., Lin Y. (2019). Soil microbial diversity during 30 years of grassland restoration on the Loess Plateau, China: Tight linkages with plant diversity. Land Degrad. Dev..

[12] Bünemann E.K., Bongiorno G., Bai Z., Creamer R.E., Brussaard L. (2018). Soil quality—A critical review. Soil Biol. Biochem..

[13] Dokuchaev V.V., Kaner N. (1967). Russian Chernozems (Russkii Chernozems).

[14] Wagg C., Schlaeppi K., Banerjee S., Kuramae E.E., van der Heijden M.G. (2019). Fungal-bacterial diversity and microbiome complexity predict ecosystem functioning. Nat. Commun..

[15] Zheng Q., Hu Y., Zhang S., Noll L., Böckle T., Dietrich M., Herbold C.W., Eichorst S.A., Woebken D., Richter A. (2019). Soil multifunctionality is affected by the soil environment and by microbial community composition and diversity. Soil Biol. Biochem..

[16] Gong X., Jarvie S., Wen J., Su N., Yan Y., Liu Q., Zhang Q. (2024). Compared with soil fungal diversity and microbial network complexity, soil bacterial diversity drives soil multifunctionality during the restoration process. J. Environ. Manag..

[17] Li J., Delgado-Baquerizo M., Wang J.-T., Hu H.-W., Cai Z.-J., Zhu Y.-N., Singh B.K. (2019). Fungal richness contributes to multifunctionality in boreal forest soil. Soil Biol. Biochem..

[18] Gao C., Xu L., Montoya L., Madera M., Hollingsworth J., Chen L., Purdom E., Singan V., Vogel J., Hutmacher R.B. (2022). Co-occurrence networks reveal more complexity than community composition in resistance and resilience of microbial communities. Nat. Commun..

[19] Kaiser K., Miehe G., Barthelmes A., Ehrmann O., Scharf A., Schult M., Schlütz F., Adamczyk S., Frenzel B. (2008). Turf-bearing topsoils on the central Tibetan Plateau, China: Pedology, botany, geochronology. Catena.

[20] Walkley A., Black I.A. (1934). An examination of the Degtjareff method for determining soil organic matter, and a proposed modification of the chromic acid titration method. Soil Sci..

[21] Vance E.D., Brookes P.C., Jenkinson D.S. (1987). An extraction method for measuring soil microbial biomass C. Soil Biol. Biochem..

[22] Meng M.-H., Liang C., He J., Shi Z.-Y., Chen F.-S., Wang F.-C., Jiang X.-L., Fang X.-M. (2025). Nutrient enrichment weakens the positive feedback of soil organic carbon decomposition to short-term warming in subtropical forests. Plant Soil.

[23] Trujillo M.E., Alonso-Vega P., Rodríguez R., Carro L., Cerda E., Alonso P., Martínez-Molina E. (2010). The genus Micromonospora is widespread in legume root nodules: The example of Lupinus angustifolius. ISME J..

[24] Quast C., Pruesse E., Yilmaz P., Gerken J., Schweer T., Yarza P., Peplies J., Glöckner F.O. (2012). The SILVA ribosomal RNA gene database project: Improved data processing and web-based tools. Nucleic Acids Res..

[25] Ding C., Liu Y., Hernández M., Sun H., Jiao S., Pan H., Ge T., Zhao K., Zhang Q., Xu J. (2025). Coupling soil bacterial and fungal community traits to multifunctionality in grassland ecosystem. Agric. Ecosyst. Environ..

[26] Maestre F.T., Quero J.L., Gotelli N.J., Escudero A., Ochoa V., Delgado-Baquerizo M., García-Gómez M., Bowker M.A., Soliveres S., Escolar C. (2012). Plant species richness and ecosystem multifunctionality in global drylands. Science.

[27] Byrnes J.E., Gamfeldt L., Isbell F., Lefcheck J.S., Griffin J.N., Hector A., Cardinale B.J., Hooper D.U., Dee L.E., Duffy J.E. (2014). Investigating the relationship between biodiversity and ecosystem multifunctionality: Challenges and solutions. Methods Ecol. Evol..

[28] Oksanen J., Blanchet F.G., Kindt R., Legendre P., Minchin P.R., O’hara R., Simpson G.L., Solymos P., Stevens M.H.H., Wagner H. (2013). Package ‘Vegan’. Community Ecology Package.

[29] Pandey B.K., Huang G., Bhosale R., Hartman S., Sturrock C.J., Jose L., Martin O.C., Karady M., Voesenek L.A., Ljung K. (2021). Plant roots sense soil compaction through restricted ethylene diffusion. Science.

[30] Shi Y., Wang Y., Ma Y., Ma W., Liang C., Flynn D., Schmid B., Fang J., He J.-S. (2014). Field-based observations of regional-scale, temporal variation in net primary production in Tibetan alpine grasslands. Biogeosciences.

[31] Yang T., Adams J.M., Shi Y., He J.s., Jing X., Chen L., Tedersoo L., Chu H. (2017). Soil fungal diversity in natural grasslands of the Tibetan Plateau: Associations with plant diversity and productivity. New Phytol..

[32] Delgado-Baquerizo M., Reich P.B., Trivedi C., Eldridge D.J., Abades S., Alfaro F.D., Bastida F., Berhe A.A., Cutler N.A., Gallardo A. (2020). Multiple elements of soil biodiversity drive ecosystem functions across biomes. Nat. Ecol. Evol..

[33] Sun J., Ma B., Lu X. (2018). Grazing enhances soil nutrient effects: Trade-offs between aboveground and belowground biomass in alpine grasslands of the Tibetan Plateau. Land Degrad. Dev..

[34] Zhang Z., Li Y., Williams R.A., Chen Y., Peng R., Liu X., Qi Y., Wang Z. (2023). Responses of soil respiration and its sensitivities to temperature and precipitation: A meta-analysis. Ecol. Inform..

[35] Tedersoo L., Bahram M., Põlme S., Kõljalg U., Yorou N.S., Wijesundera R., Ruiz L.V., Vasco-Palacios A.M., Thu P.Q., Suija A. (2014). Global diversity and geography of soil fungi. Science.

[36] Bahram M., Hildebrand F., Forslund S.K., Anderson J.L., Soudzilovskaia N.A., Bodegom P.M., Bengtsson-Palme J., Anslan S., Coelho L.P., Harend H.J.N. (2018). Structure and function of the global topsoil microbiome. Nature.

[37] Guo X., Feng J., Shi Z., Zhou X., Yuan M., Tao X., Hale L., Yuan T., Wang J., Qin Y. (2018). Climate warming leads to divergent succession of grassland microbial communities. Nat. Clim. Chang..

[38] Zhang J., Wang F., Che R., Wang P., Liu H., Ji B., Cui X. (2016). Precipitation shapes communities of arbuscular mycorrhizal fungi in Tibetan alpine steppe. Sci. Rep..

[39] Ma L., Zhang C., Xu X., Wang C., Liu G., Liang C., Zuo X., Wang C., Lv Y., Wang R. (2022). Different facets of bacterial and fungal communities drive soil multifunctionality in grasslands spanning a 3500 km transect. Funct. Ecol..

[40] Buckeridge K.M., Mason K.E., Mcnamara N.P., Ostle N., Puissant J., Goodall T., Griffiths R.I., Stott A.W., Whitaker J. (2020). Environmental and microbial controls on microbial necromass recycling, an important precursor for soil carbon stabilization. Commun. Earth Environ..

[41] Crowther T.W., Sokol N.W., Oldfield E.E., Maynard D.S., Thomas S.M., Bradford M.A. (2015). Environmental stress response limits microbial necromass contributions to soil organic carbon. Soil Biol. Biochem..

[42] Han X., Doménech-Pascual A., Donhauser J., Zohner C.M., Mo L., Crowther T.W., Casas-Ruiz J.P., Jordaan K., Ramond J.-B., Romaní A.M. (2025). Fungal diversity as a key driver of soil multifunctionality along a European latitudinal gradient. Geoderma.

[43] Eastwood D.C., Floudas D., Binder M., Majcherczyk A., Schneider P., Aerts A., Asiegbu F.O., Baker S.E., Barry K., Bendiksby M. (2011). The plant cell wall–decomposing machinery underlies the functional diversity of forest fungi. Science.

[44] Šnajdr J., Valášková V., Merhautová V.R., Herinková J., Cajthaml T., Baldrian P. (2008). Spatial variability of enzyme activities and microbial biomass in the upper layers of Quercus petraea forest soil. Soil Biol. Biochem..

[45] Baldrian P. (2017). Forest microbiome: Diversity, complexity and dynamics. FEMS Microbiol. Lett..

[46] Prendergast-Miller M.T., Baggs E.M., Johnson D. (2011). Nitrous oxide production by the ectomycorrhizal fungi Paxillus involutus and Tylospora fibrillosa. FEMS Microbiol. Lett..

[47] Liu X., Chu H., Godoy O., Fan K., Gao G.-F., Yang T., Ma Y., Delgado-Baquerizo M. (2024). Positive associations fuel soil biodiversity and ecological networks worldwide. Proc. Natl. Acad. Sci. USA.

