# Supplementary material for: Fungal Diversity Drives Non-Linear Trajectories of Soil Multifunctionality During Alpine Grassland Restoration
Source: Microorganisms. 2026 Mar 1;14(3):562. doi: 10.3390/microorganisms14030562 (PMC13029105; doi:10.3390/microorganisms14030562)
Supplement: Supplementary file 1 [file microorganisms-14-00562-s001.zip › microorganisms-4146420-supplementary.pdf]

Fungal Diversity Drives Non-linear Trajectories of Soil Multifunctionality during Alpine Grassland Restoration

Minghui Meng[1,2,†], Jiakai Shi[1,2,†], Sha Zhou[3], Danni Peng[1,2], Yihan Fu[1,2], Mengmeng Wen[1,2], Jun Wang[1,2,4,5], Fazhu Zhao[1,2,4,6,*]

[1] College of Urban and Environmental Sciences, Northwest University, Xi'an, Shaanxi 710127, China mmh19990810@163.com (M.M.)
[2] Shaanxi Key Laboratory of Earth Surface System and Environmental Carrying Capacity, Northwest University, Xi'an, Shaanxi 710127, China
[3] Xi'an Botanical Garden of Shaanxi Province (Institute of Botany of Shaanxi Province), Xi'an, China
[4] Carbon Neutrality College (Yulin), Northwest University Xi'an, Xi'an, Shaanxi 719053, China
[5] State Key Laboratory of Soil Erosion and Dryland Farming on the Loess Plateau, Institute of Soil and Water Conservation, Chinese Academy of Sciences and Ministry of Water Resources, Yangling, China
[6] Shaanxi Xi'an Urban Ecosystem National Observation and Research Station, National Forestry and Grassland Administration, Xi'an, China
[†] These authors contributed equally to this work

* Correspondence: zhaofazhu@nwu.edu.cn

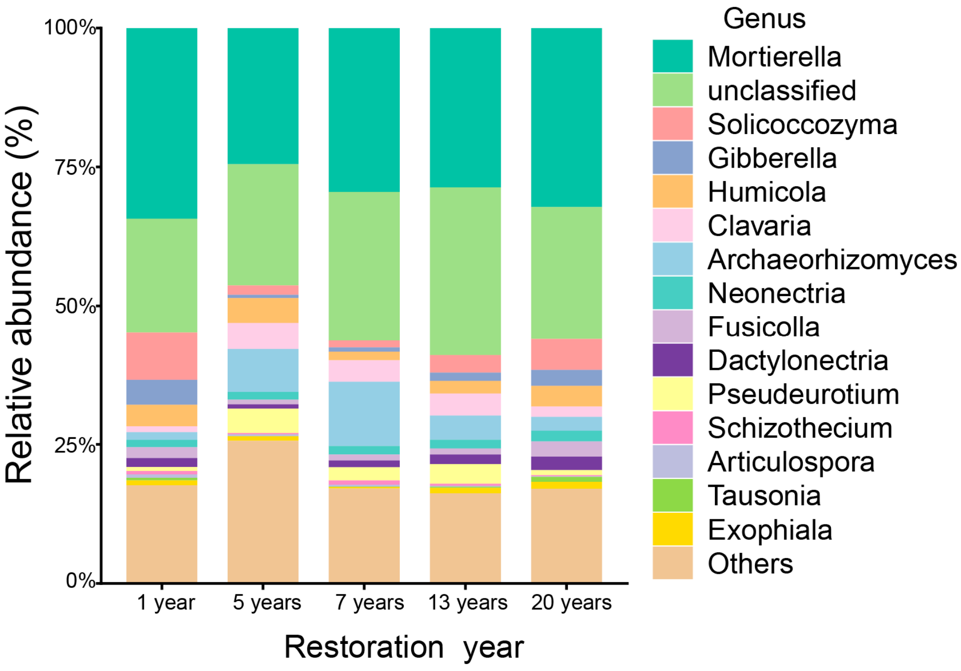

Figure S1. Relative abundance of fungal taxa across the restoration chronosequence.

a

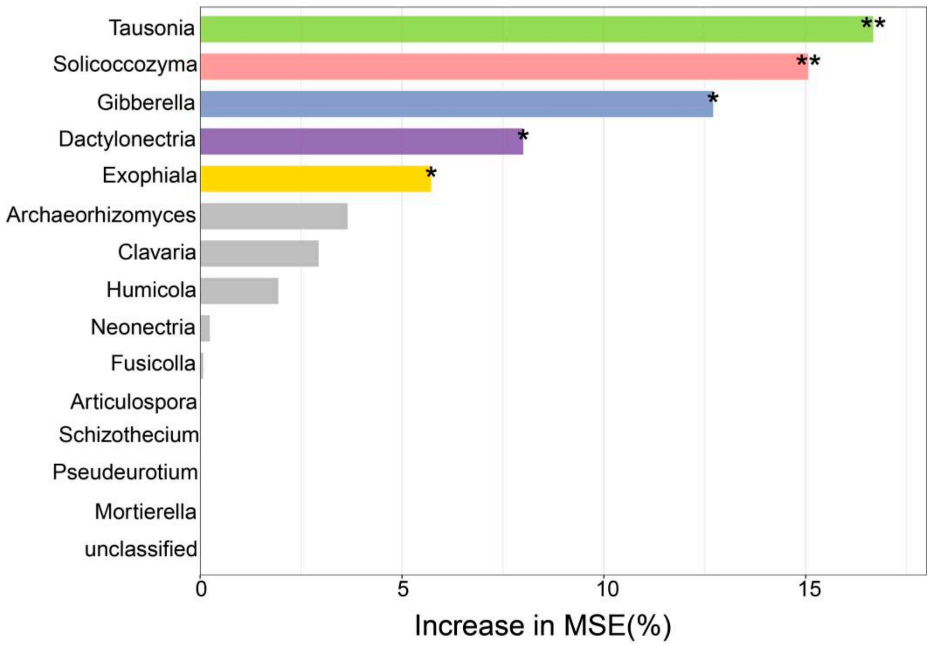

b

Genus	ρ	Trophic Mode
Tausonia	-0.722**	Saprotroph
Solicoccozyma	-0.539**	Saprotroph-Symbiotroph
Gibberella	-0.457*	Pathotroph-Saprotroph-Symbiotroph
Dactylonectria	-0.612*	Pathotroph
Exophiala	-0.419*	Pathotroph-Saprotroph-Symbiotroph

Figure S2. Importance of fungal taxa at the genus level identified as predictors of soil multifunctionality using random forest modeling. Panel (a) shows the mean predictor importance (% of increase of MSE) of fungal genera on soil multifunctionality, based on the random forest model. Panel (b) shows the correlations between the significant fungal genera and soil multifunctionality. Significance levels are indicated as follows: * $P < 0.05$; ** $P < 0.01$; *** $P < 0.001$.

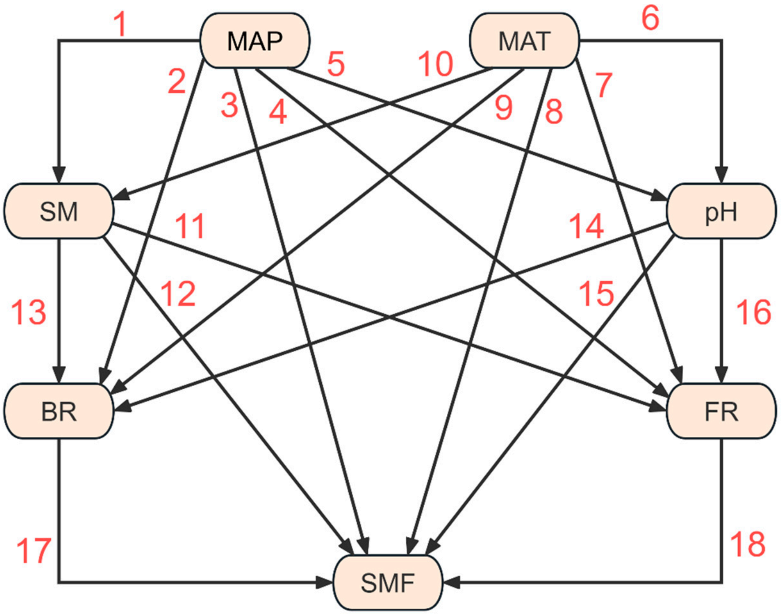

#	Associations	Rationale	Ref.
1	MAP→SM	The joint distributions of precipitation and soil moisture were analyzed at monthly and annual scales, using soil moisture and precipitation data from ERA5-Land and Global Precipitation Climatology Project, respectively. The nonlinear negative dependences reached to 19.2 %, 0.7 %, and 2.3 % at monthly scale.	(Xue et al., 2025)
2,4,7,9	MAP, MAT →BR, FR	It has been well documented that soil microbial diversity is shaped by a range of factors, including climate (temperature and precipitation), land cover, space (latitude and longitude), and edaphic variables (pH, texture, nutrients, organic matter, etc.)	(Bahram et al., 2018; Labouyrie et al., 2023; Liu et al., 2020).
3	MAP→SMF	Climatic and other environmental differences among sites, such as MAP, may also be key drivers in shaping the positive relationship between biodiversity and EMF as well as the differences between aboveand belowground communities.	(Ma et al., 2010)
5	MAP→ pH	MAP was the main factor affecting	(Xie et al., 2022)

		soil pH (r= −0.7244). In the regions with MAP > 800 mm, soil pH was negatively correlated with MAP (r= −0.6651).	
6	MAT→ pH	The results showed that soil pH decreased along the gradient of both mean annual temperature and precipitation.	(Chen et al.,2014)
8	MAT→SMF	Mean annual temperature generates strong negative effects on BEMF in MAT ≤ 16.4 °C regions, whereas precipitation and plant species richness positively dominate the dynamics of BEMF in regions where MAT > 16.4 °C.	(Zhou et al., 2025)
10	MAT→SM	These findings suggest that further increases in mean annual temperature and evapotranspiration may lead to decreases in soil moisture.	(Dymond et al., 2014)
11,13,14,16	SM, pH→ FR, BR	Soil pH and moisture were the two main factors that significantly affected bacterial and fungal diversity.	(Duan et al., 2023)
12	SM→SMF	We found that soil moisture was the most important driver, accounting for 65% of the variation in EMF.	(Jing et al., 2015)
15	pH→ SMF	For instance, pH can largely influence the rates of ecosystem processes as enzymes.	(Hu et al., 2021)
17,18	FR, BR→SMF	Soil microbial diversity can affect ecosystem processes and energy flow.	(Sokol et al., 2022)

Figure S3. A priori structural equation modeling (SEM) metamodel evaluating the drivers of soil multifunctionality.

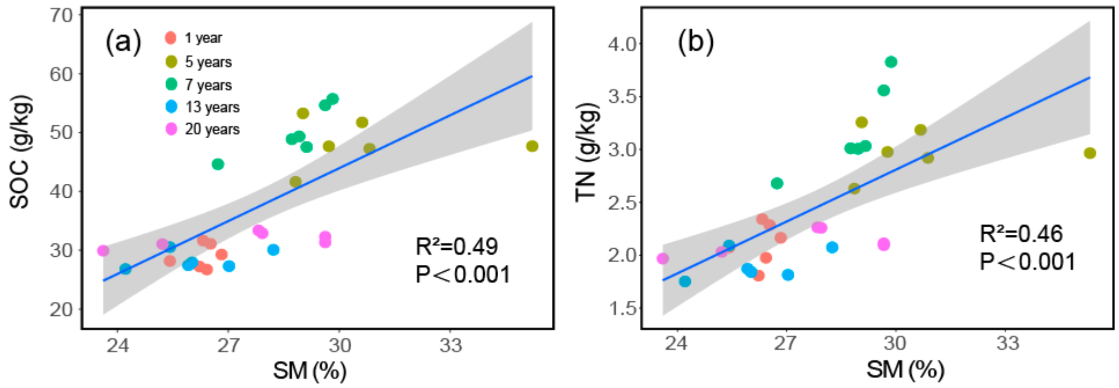

Figure S4. Correlations between soil moisture and soil organic carbon (a), total nitrogen (b).

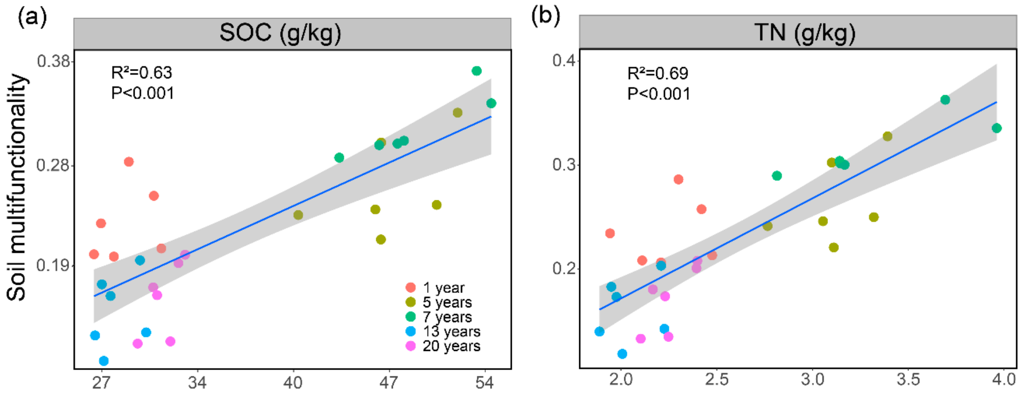

Figure S5. Correlations between soil multifunctionality and soil organic carbon (a), total nitrogen (b).

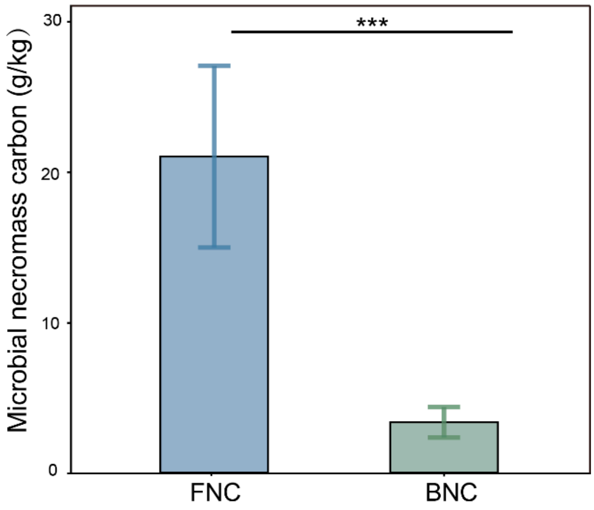

Figure S6. Differences in fungal and bacterial necromass carbon. Significant differences are indicated by asterisks (***$P < 0.001$). FNC: fungal necromass carbon; BNC: bacterial necromass carbon.

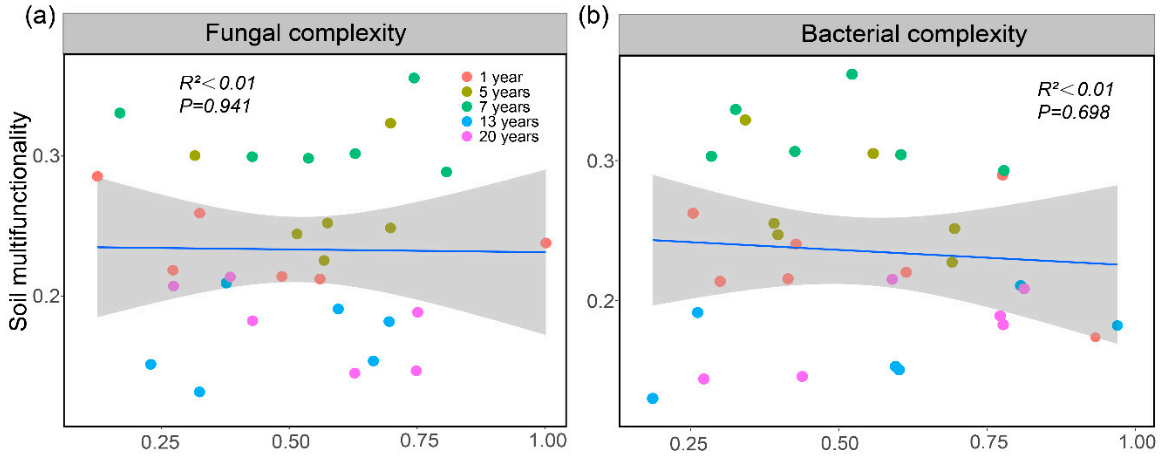

Figure S7. Correlations between soil multifunctionality and fungal (a) or bacterial complexity (b).

Table S1. Dominant plant species in the alpine grassland study area

Scientific name	Family	Genus	Life form
Kobresia pygmaea	Cyperaceae	*Kobresia*	Perennial herb
Oxytropis ochrocephala	Fabaceae	*Oxytropis*	Perennial herb
Carex moorcroftii	Cyperaceae	*Carex*	Perennial herb
Elsholtzia densa Benth	Lamiaceae	*Elsholtzia*	Annual herb
Kobresia humilis	Cyperaceae	*Kobresia*	Perennial herb
Elymus nutans	Poaceae	*Elymus*	Perennial herb
Stipa purpurea	Poaceae	*Stipa*	Perennial herb
Poa spp.	Poaceae	*Poa*	Perennial herb
Potentilla anserina	Rosaceae	*Potentilla*	Perennial herb
Potentilla bifurca	Rosaceae	*Potentilla*	Perennial herb
Pedicularis kansuensis	Orobanchaceae	*Pedicularis*	Perennial herb
Anaphalis lactea	Asteraceae	*Anaphalis*	Perennial herb
Taraxacum mongolicum	Asteraceae	*Taraxacum*	Perennial herb
Thalictrum alpinum	Ranunculaceae	*Thalictrum*	Perennial herb

Table S2. Spearman correlations among soil variables used to calculate the soil multifunctionality index. Correlation coefficients and their significance levels are shown. **P*<0.05, ***P*<0.01 and ****P*<0.001.

	**POC**	**MAOC**	**TP**	**AP**	**NH_4-N**	**MBC**	**MBN**	**MBP**	**DON**	**DOC**	**PER**	**PPO**	**NAG**	**URE**
POC		0.29	0.20	0.17	0.25	0.61***	-0.02	0.51**	0.33	0.17	-0.37*	-0.25	0.64***	0.29
MAOC			0.41*	0.39*	0.07	0.37*	0.09	0.02	0.21	0.22	-0.17	-0.14	0.63***	0.04
TP				0.67***	-0.05	0.59***	-0.13	0.22	-0.31	0.10	0.13	0.13	0.31	0.40*
AP					-0.20	0.30	-0.10	0.20	0.12	0.05	-0.12	0	0.28	0.16
NH_4-N						0.18	0.03	-0.06	0.21	0.32	0.04	0.11	0.04	0.15
MBC							-0.04	0.49**	-0.03	0.19	0.09	0.02	0.39*	0.41*
MBN								0.12	0.23	-0.13	-0.16	-0.13	-0.20	-0.02
MBP									0.03	-0.06	0.10	0.07	0.16	0.37*
DON										-0.03	-0.44*	-0.44*	0.35	-0.38*
DOC											0.39*	0.34	-0.03	0.58***
PER												0.46**	-0.48**	0.31
PPO													-0.45*	0.44*
NAG														-0.20
URE														

Table S3. The basic soil properties during grassland restoration. Different letters indicate significant differences among stages (one-way ANOVA followed by LSD, $P < 0.05$). Values are means ± SD (n = 6).

**Parameters**	**Unit**	**1 year**	**5 years**	**7 years**	**13 years**	**20 years**
SOC	g/kg	28.83±2.02b	48.00±4.06a	49.92±4.26a	28.15±1.56b	31.60±1.28b
TN	g/kg	2.24±0.20b	3.12±0.22a	3.32±0.42a	2.04±0.14b	2.26±0.12b
SM	%	26.27±0.47b	30.68±2.4a	28.80±1.11ab	26.12±1.37b	27.28±2.42b
SWC	%	24.49±2.28b	40.97±2.84a	45.98±4.89a	29.42±1.49b	29.99±0.85b
SOC/TN	-	12.87±0.51c	15.35±0.23a	15.10±0.66ab	13.79±0.25c	14.01±0.25bc
Vegetation Cover	%	74.67±6.31b	90.83±6.27a	98.83±1.75a	92.83±3.19a	91.67±3.39a

Table S4. Topological properties of fungal co-occurrence networks across the restoration chronosequence. Different letters indicate significant differences among stages (one-way ANOVA followed by LSD, $P < 0.05$). Values are means ± SD (n = 6).

Fungi network topology properties	1 year	5 years	7 years	13 years	20 years
Nodes	421b	462a	425b	421b	408b
Edges	1730b	3019a	2262b	2065b	1668b
Average degree	8.22c	13.06a	10.59b	9.79bc	8.17c
Modularity	0.69b	0.73a	0.73a	0.70ab	0.72a
Graph density	0.02c	0.03a	0.02ab	0.023bc	0.020c
Clustering coefficient	0.52d	0.68a	0.63ab	0.60bc	0.56cd
Betweenness centrality	0.037a	0.05a	0.04a	0.05a	0.04a
